# Efficacy of zinc sulfate on concurrent chemoradiotherapy induced taste alterations in oral cancer patients: A double blind randomized controlled trial

**DOI:** 10.12669/pjms.35.3.503

**Published:** 2019

**Authors:** Asma Hayat Khan, Jawad Safdar, Saad Uddin Siddiqui

**Affiliations:** 1 *Dr. Asma Hayat Khan, BDS, MSc (PG), Dow University of Health Sciences, Karachi, Pakistan*; 2 *Dr. Jawad Safdar, BDS, MDS, PhD, Assistant Professor OMFS, Dow University of Health Sciences, Karachi, Pakistan*; 3 *Dr. Saad Uddin Siddiqui, BDS, M.O.M.S (RCSED), M.F.D.S (RCPSG) Senior Registrar Oral Medicine, Dow University of Health Sciences, Karachi, Pakistan*

**Keywords:** Concurrent chemo-radiotherapy (CCRT), Oral cancer, Taste acuity, Zinc sulfate, **CCRT =** Concurrent chemoradiotherapy, **DT =** Detection threshold, **RT =** Recognition threshold, **AEMC =** Atomic Energy Medical Centre

## Abstract

**Objectives:**

To observe the efficacy of zinc sulfate on taste alterations in oral cancer patients receiving concurrent chemotherapy with radiotherapy.

**Methods:**

Seventy patients were randomly assigned to both intervention and control group at Oncology Section of Atomic Energy Medical Centre Karachi from September 2017 to March 2018. One group received zinc sulfate capsules (50 mg TDS daily after meals) and the other group received placebo (thrice after meals). Patients were advised to start taking capsules on the first day of their chemoradiation. Both the groups continued the capsules a month after their CCRT ended.

**Results:**

Sweet taste was most effected by cancer and its treatment followed by bitter and salty taste. Sour taste was least effected. When both the groups were compared for four tastes for detection threshold, the differences in observation at 3 stages of median IQR were not significant. For recognition threshold between zinc sulfate and placebo, no significant difference was observed in median IQR for salty taste and bitter taste. However, sweet taste (baseline p-value 0.245, end p-value 0.010, follow-up p-value 0.038) was statistically significant at end of CCRT and follow-up stage and sour taste (baseline p-value 0.24, end p-value 0.006, follow-up p-value 0.898) at end of CCRT only.

**Conclusion:**

Zinc sulfate was not found to be beneficial in preventing chemoradiation induced taste alterations. Taste and smell alterations are common in patients with cancer and do not receive sufficient support to manage taste alterations. This area requires more research to develop a comprehensive understanding of the nature and its management.

## INTRODUCTION

Taste changes are common in cancer patients receiving concurrent chemoradiation which become a significant complaint and a cause of distress and morbidity. Taste is an essential sensation which serves oral intake of food and enables to prevent the ingestion of potentially harmful and poisonous substances.^1^ The sense of taste is fundamental to sustain health and quality of life and is crucial for an individual’s well-being and psychological health, yet it is generally taken for granted. Taste disorders can range from distortion of taste to reduced ability to taste to complete absence of taste.^2^ Taste impairment is common in cancer patients which may be due to the disease itself or because of the cancer treatments.^3^ The prevalence of taste dysfunction in cancer patients has been reported to be up to 77%.^4^ Reduction in taste acuity occurs due to direct damage to the taste buds or as a result of changes in the saliva during chemoradiation of the oral cancer.^5^ Taste changes may advance to reduced appetite, dietary insufficiency, food repulsion affecting body weight and anorexia further leading to impaired immunity, decline in health status and malnutrition.^6^ Malnutrition in extreme cases can have a detrimental impact on life and reduce patient outcomes and is accountable for death in up to 20% of cancer patients.^7^ Taste alterations are understudied and underestimated despite being the commonest side effect of CCRT.

Zinc is a vital micronutrient and is one of the most copious elements in the human body and, unlike iron, it has no storage site in the human body. It is comparatively non-toxic if taken orally.^8^ Zinc has been in spotlight over recent years due to its antioxidant properties and biochemical processes and homeostatic functions. Zinc is an integral element in both the maintenance and repair of taste buds. Zinc is an important cofactor for alkaline phosphatase and is needed for its synthesis; the most important and abundant enzyme in taste bud membranes.^2^ Zinc influences the synthesis of gustin (carbonic anhydrase VI) required for the growth, development, maintenance and production of taste buds and regulation of taste function.^9^ Taste buds depend on calcium receptors to work appropriately. Zinc is also believed to be responsible for raising calcium concentration in saliva.^10^ Our objective was to observe the efficacy of zinc sulfate on taste alterations in oral cancer patients receiving concurrent chemotherapy with radiotherapy

## METHODS

Data was collected from patients visiting the Oncology Section at Atomic Energy Medical Centre Karachi from September October 2017 to March 2018. After taking written informed consent, patients were recruited into test and control group by Random Allocation Sequence. The study was ethically approved by Institution Review Board of Dow University of Health Sciences, Karachi. The trial was registered on ClinicalTrials.gov NCT03824925. All the patients with histopathologically proven oral cancer who were about to start concurrent chemotherapy with radiotherapy (CCRT) as a single treatment modality for the first time, both gender aged between 20 to 60 years with radiation planned between 60-70 Gy of external beam radiotherapy and Cisplatin given as primary chemotherapeutic agent were included. Exclusion criteria were previous history of radiotherapy or chemotherapy, existence of oral lesions such as aphthous ulcers, stomatitis or candidosis at the time of selection, cranial nerve lesions of V, VII, IX and partial or total glossectomy, individuals with nose or ear infections which can influence taste, metabolic or endocrine disorders that may affect taste sensitivity (Sjogren syndrome, hypertension, diabetes mellitus, renal disease, liver disease and thyroid disease), concomitant administration of a drug with CCRT which may affect taste (metronidazole, diuretics and anti-depressants), individuals with already on medications such as penicillamine, tetracyclines, quinolones, bisphosphonates for any existing condition, patients who didn’t agree to participate and sign consent form and lack of cooperation.

All the taste solutions for assessing taste acuity were made in accordance to ISO 3972:2011/Cor 1:2012.^11^ Patients taste acuity was observed by detection and recognition threshold through different concentration of taste solutions for each taste at three stages of observation that is, baseline, end CCRT (approximately after 7-8 weeks from the start of treatment) and follow-up after a month (11-12 weeks approx.). Detection threshold (DT) was considered as the lowest level at which a subject can perceive a stimulus. This was the minimum concentration at which the subject can detect that there is something different from water, but may not identify its quality.^12^ Patients were also asked to describe the taste of the solution either salty, sweet, sour or bitter. Recognition threshold was considered as the lowest level of a solution at which a subject can correctly recognize its taste.^12^,^13^ D1 was the highest concentration of solution for each taste whereas D8 was the minimum concentration and was marked as 8.

- Cut-off value set for recognition threshold was;
- 1-8; able to detect and recognize the taste correctly.
- 0; unable to recognize the any taste or incorrectly identifies it.

After measuring taste acuity before chemoradiotherapy (at baseline), one group was given zinc sulfate capsules 50 mg and the other group received placebo. Both groups were advised to take the capsule thrice a day after meals with a full glass of water. Compliance of the patients was monitored by counting monthly pills. The treatment lasted for almost three months. Patients were advised not to take any concurrent zinc supplement simultaneously. Zinc sulfate was custom made by ATCO pharmaceuticals as Zincat.

Each patient’s chemotherapy and radiotherapy were started at the same day. Cisplatin 50mg intravenously was given to the patients weekly until the end of their radiotherapy. Only two patients could not tolerate Cisplatin and therefore were switched to Carboplatin 50mg IV weekly. The standard dose of radiotherapy given to oral cancer patients at AEMC is 66 Gy, given in 35 fractions with 2 Gy of daily fraction for consecutive 5 days (rest of 2 days), over a period of 7-8 weeks. Thus, the radiotherapy dose was same for the patients in both groups. The entire neck, including the supraclavicular areas and the posterior neck, was irradiated and off cord at 50 Gy.

### Statistical analysis

Data was analyzed by SPSS software 20. Data is presented as median [IQR] for intervention statistics. The normality was assessed of all four taste variables by Shapiro-Wilk test. The baseline characteristics of the test and control group were compared by Chi-square and independent t-test. To observe the differences between the two groups, Mann-Whitney U test was used. P-value was calculated by Friedman test. Statistical significance was accepted at P < 0.05.

## RESULTS

Of 70 patients, 68 completed the trial. Patients were randomized into zinc intervention (mean age 43.4 ± 12, 23.5% females) and placebo group (mean age 46 ± 9.2, 32.4% females). The two groups were similar with respect to the baseline characteristics (Table I-V). Compliance was favorable throughout the study.

**Table-I:** Demographic and clinical characteristics of patients in zinc and control group.

	Placebo	Zinc	P-value
	n=34 (%)	n=34 (%)
***Age (years)***			
Mean ± SD	46.03±9.22	43.44±12.01	0.323*
***Weight (Kg)***			
Mean ± SD	60.38±15.01	58.88±12.35	0.654*
***Gender***			
Male	23(67.6)	26(76.5)	0.417**
Female	11(32.4)	8(23.5)
***Tumor Site***			
Buccal mucosa	24(70.6)	19(55.9)	0.194**
Dorsum tongue	8(23.5)	12(35.3)	
Base – tongue	2(5.9)	0	
Others	0	3(8.8)
***Tumor Stage***			
I	1(2.9)	2(5.9)	N/A
II	11(32.4)	13(38.2)	
III	11(32.4)	9(26.5)	
IV	11(32.4)	10(29.4)	

* Independent t test,

** Chi-square, N/A= Not applicable due to cells have expected count less than 5, level of Significance 0.05

**Table-II:** Detection and Recognition threshold.

Group (DT)	Placebo (n =34)		Zinc (n =34)		Test	

Salty Taste	Median	IQR	Median	IQR	Mann-Whitney U	P-value
Baseline	7.00	(8.00-6.00)	7.00	(8.00-5.75)	575.00	0.97
End CCRT	6.00	(7.00-5.00)	6.00	(7.00-5.00)	517.00	0.44
Follow-up	7.00	(7.00-5.50)	7.00	(7.25-6.00)	459.50	0.12

*Group (RT)*	*Placebo (n =34)*		*Zinc (n =34)*		*Test*	

*Salty Taste*	*Median*	*IQR*	*Median*	*IQR*	*Mann-Whitney U*	*P-value*

Baseline	6.00	(7.00-4.75)	6.00	(7.00-0.75)	506.50	0.37
End CCRT	5.00	(5.25-1.75)	5.00	(6.00-4.00)	484.0	0.24
Follow-up	6.00	(7.00-1.00)	6.00	(7.00-4.00)	541.5	0.65

*level of Significance 0.05

**Table-III:** Detection and Recognition threshold

Group (DT)	Placebo (n =34)		Zinc (n =34)		Test	

Sweet Taste	Median	IQR	Median	IQR	Mann-Whitney U	P-value
Baseline	5.50	(7.25-3.75)	5.50	(7.00-2.50)	521.00	0.48
End CCRT	5.50	(6.00-4.00)	5.00	(7.00-3.75)	571.00	0.93
Follow-up	5.50	(6.00-4.00)	6.00	(6.25-4.50)	505.50	0.36

*Group (RT)*	*Placebo (n =34)*		*Zinc (n =34)*		*Test*	

*Sweet Taste*	*Median*	*IQR*	*Median*	*IQR*	*Mann-Whitney U*	*P-value*
Baseline	5.00	(6.25-3.00)	4.00	(6.00-1.00)	484.0	0.25
End CCRT	3.00	(4.00-1.00)	5.00	(6.00-3.00)	371.50	0.01*
Follow-up	3.50	(5.00-1.00)	5.00	(6.00-2.75)	411.50	0.04*

* level of Significance 0.05

**Table-IV:** Detection and Recognition threshold.

Group (DT)	Placebo (n =34)		Zinc (n =34)		Test	

Sour Taste	Median	IQR	Median	IQR	Mann-Whitney U	P-value
Baseline	8.00	(8.00-6.00)	8.00	(8.00-7.00)	486.00	0.20
End CCRT	6.50	(7.00-5.00)	7.00	(7.00-6.00)	442.00	0.07
Follow-up	7.00	(7.25-6.00)	7.00	(8.00-6.00)	547.00	0.69

*Group (RT)*	*Placebo (n =34)*		*Zinc (n =34)*		*Test*	

*Sour Taste*	*Median*	*IQR*	*Median*	*IQR*	*Mann-Whitney U*	*P-value*

Baseline	7.00	(8.00-5.75)	8.00	(8.00-5.75)	486.50	0.24
End CCRT	5.50	(6.00-5.00)	6.00	(7.00-6.00)	365.0	0.006*
Follow-up	7.00	(7.00-5.75)	6.00	(7.00-6.00)	5680	0.89

* level of Significance 0.05

**Table-V:** Detection and Recognition threshold.

Group (DT)	Placebo (n =34)		Zinc (n =34)		Test	

Bitter Taste	Median	IQR	Median	IQR	Mann-Whitney U	P-value
Baseline	6.00	(8.00-5.00)	7.00	(8.00-5.00)	563.500	0.856
End CCRT	6.00	(7.00-3.75)	5.50	(7.00-5.00)	558.500	0.808
Follow-up	6.00	(7.00-5.00)	6.00	(7.00-6.00)	441.000	0.084

*Group (RT)*	*Placebo (n =34)*		*Zinc (n =34)*		*Test*	

*Bitter Taste*	*Median*	*IQR*	*Median*	*IQR*	*Mann-Whitney U*	*P-value*
Baseline	6.00	(7.00-5.00)	5.00	(7.00-0.50)	474.5	0.197
End CCRT	4.00	(6.00-1.75)	4.00	(5.00-0.75)	487.5	0.26
Follow-up	5.00	(6.00-3.00)	5.50	(6.00-1.75)	526.5	0.518

*level of Significance 0.05

### Difference of median (IQR) between placebo and zinc group for four basic tastes

### Salty taste

No statistical difference was observed between both the groups in DT and RT.

### Sweet taste

Slight improvement in DT was observed in zinc group i.e. lower concentrations of sweet taste was required to detect it being different from water. However, the difference was not statistically significant. In RT, patients in zinc group showed improvement in their taste acuity and the difference was statistically significant.

### Sour taste

In DT, slight improvement was observed in zinc group but the difference was not statistically significant. In RT, statistically significant difference was observed during end of CCRT.

### Bitter taste

Improvement in DT and RT was observed in patients taking zinc supplements however the difference was not statistically significant between both the groups.

## DISCUSSION

To our knowledge, this is for the very first time in our region that taste alterations in oral cancer patients undergoing CCRT were studied. In present trial, zinc slightly improved taste acuity in patients taking zinc supplement however the difference was not statistically significant between both the groups. The predicted reason for the results of present study to differ from the previous studies was the treatment modality selected for oral cancer patients and methodology. Previous trails observed the effect of zinc on taste in either radiotherapy or chemotherapy alone. CCRT has emerged as an acceptable therapy for oral cancers because of its good survival rate.^14^ Although CCRT means combining their adverse effects which leads to acute toxicity which might had surpassed the beneficial effect of zinc sulfate on taste acuity. The other factor was the methodology used in present trial to assess the taste changes. The present study used ISO Method of investigating sensitivity of taste which helped in generating meaningful data.

The results of the present study were supported by an experimental trial by Halyard et al. who performed a double-blind randomized controlled trial. No preventive effect of zinc was observed in patients who received RT for H&N cancer.^15^ Henkin et al. in a double-blind cross-over trial observed no significant difference between zinc and control group.^16^ Contrary to results of present trial, Ripamonti et al. detected a slower worsening of the taste and faster recovery of the taste function in patients who received zinc sulfate thrice daily.^17^ Najafizade et al administered 50mg zinc sulfate thrice daily to patients who received RT and observed preventive effects of zinc sulfate on radiation-induced taste alterations.^18^ Three-drop method was used by Najafizade to measure taste acuity in which three drops of each taste solution were poured on patients’ tongue which might not have exposed all the taste buds in oral cavity leading to selective detection and recognition. However, the present trial used sip and spit method and our sample size was much bigger. According to present trial, sweet taste was most disturbed in both zinc and placebo at all three stages followed by bitter and salty taste. Bitter taste was found to be most effected in other studies.^19^,^20^

Studies support the fact that exogenous zinc might be beneficial in regulating taste function in zinc deficient subjects or idiopathic taste dysfunction.^21^ Oral zinc sulfate was not found to cause any adverse effects in previous randomized trials.^22^ Similarly in the present study also none of the patient reported any adverse effect of zinc such as abdominal pain or vomiting. Prophylactic administration of zinc or use of amifostine in the treatment of dysgeusia has limited benefits and nutritional counselling is emphasized to help with dysgeusia.^23^

Individuals mostly depend on vision and hearing and increasingly touch but unfortunately taste and smell are largely under-investigated and under-explored areas. Altered taste and smell can negatively influence nutritional status. Taste dysfunction is a potentially serious adverse effect as it leads to compromised nutritional status, weight loss and psychological well-being.^24^ Dry mouth and mucositis, in addition, make eating a challenge and further limits food options. Emotional and social impacts have also been documented in cancer patients suffering from taste disorders. As taste impairment is not a life threatening event therefore it might not be reported by some patients.^25^ Therefore, emphasis must be laid on dietary counselling and meal intake should be encouraged with food of pleasant taste, color and smell and suggestion of food aromas. Thus, dietary counselling is essential to provide a basic meal plan with additional food supplements to be initiated at the beginning of the treatment and followed with some modifications until few months after the therapy has ended. Therefore, a multimodal approach is recommended by a multidisciplinary team to help patients manage the symptoms and consequences of post-treatment of oral cancer.

### Limitations of the study

The subjects were not followed-up for long. Hence, long-term effects and safety of zinc therapy could not be studied. Strict exclusion criteria in our study also made selection of the patients difficult. Serum zinc levels were not measured. We only included patients with oral cavity cancer however, taste alterations are also seen in other cancers as well.

## CONCLUSION

Based on the present study, zinc sulfate was not significantly beneficial in preventing chemoradiation induced taste alterations. Until another therapy is identified, it seems that the best approach to deal taste changes with practical measures which includes oral hygiene measures and choosing of appealing foods.

### Author`s Contribution

**AHK:** Conceptualized the idea, designed the study, collected patient data, wrote manuscript and did statistical analysis.

**JS:** Did review and final approval of the manuscript.

**SUS:** Did statistical analysis and editing of the manuscript.
